# The UV-Visible Absorption Spectra of Coumarin and Nile Red in Aqueous Solution: A Polarizable QM/MM Study

**DOI:** 10.3390/molecules30244675

**Published:** 2025-12-05

**Authors:** Tommaso Giovannini, Matteo Ambrosetti, Chiara Cappelli

**Affiliations:** 1 Department of Physics, University of Rome Tor Vergata, Via della Ricerca Scientifica 1, I-00133 Rome, Italy; 2 Classe di Scienze, Scuola Normale Superiore, Piazza dei Cavalieri 7, I-56126 Pisa, Italy

**Keywords:** polarizable QM/MM, solvatochromism, UV-Vis absorption, hydrogen bonding, water, coumarin, nile red

## Abstract

We present a comprehensive computational study of the UV-visible absorption spectra of 7-methoxycoumarin and Nile red in aqueous solution. Our fully atomistic workflow couples classical molecular dynamics (MD) with polarizable QM/MM based on fluctuating charges (QM/FQ) and dipoles (QM/FQFμ). Ensemble-averaged spectra are constructed from the snapshots extracted from the MD, embedding solvent fluctuations and specific solute–solvent interactions in the electronic response of organic dyes. The spectral profiles, obtained at the various levels, reflect the underlying solute–solvent interactions and dynamics, and we rationalize them in terms of hydrogen bonding and frontier molecular orbitals involved in the main electronic transitions. Finally, the simulated spectra and solvatochromic shifts are compared with the available experimental data, showing an overall good agreement and demonstrating the robustness of the computational protocol.

## 1. Introduction

The UV-Visible spectroscopy is a widely used instrumental technique for identifying and analyzing molecular systems in solution [1,2,3,4,5,6]. It is exploited to characterize molecular systems through their electronic transitions, which are shaped by molecular structure, dynamics, and interactions with the surrounding environment. The technique can be applied to probe complex mixtures, reaction kinetics, and it is a powerful tool for monitoring the stability and degradation of compounds over time [6,7]. In this context, theoretical and computational investigations of absorption spectra are crucial for elucidating molecular behavior and intermolecular interactions [8,9,10,11,12,13]. Theoretical approaches can in fact predict the electronic and structural features, assisting the interpretation of experimental measurements and guiding the design of compounds with targeted physicochemical properties [3,5,13,14,15].

However, the UV-Vis spectra can be strongly influenced by the environment, particularly solvents [1]. The common strategies to capture solvent effects on the electronic structure employ multiscale implicit or explicit models: the solute is treated at the quantum-mechanical (QM) level, while the environment is described more approximately, under the assumption that it modulates—but does not solely determine—the measured spectrum [16,17,18,19,20,21,22,23]. For systems with strong, specific solute–solvent interactions (e.g., hydrogen bonding), fully atomistic QM/Molecular Mechanics (QM/MM) schemes have become standard [24,25,26,27,28,29,30,31,32,33]. These approaches provide a detailed, dynamic picture of solute–solvent coupling, especially when mutual polarization is included through polarizable QM/MM [29,34,35,36,37,38,39], achieving accurate yet cost-effective descriptions and overcoming limitations of non-polarizable variants [12,40].

In this study, we employ two fully polarizable multiscale schemes, QM/Fluctuating Charges (QM/FQ [33]) and QM/Fluctuating Charges and Dipoles (QM/FQFμ [41]), to investigate the UV-Vis spectra of organic dyes in water. In QM/FQ, the MM region is endowed with fluctuating charges [33], whereas QM/FQFμ also includes an additional set of fluctuating dipoles [41]. The charges (and dipoles) adapt to the QM electrostatic potential (and field) and are then introduced into the molecular Hamiltonian, thereby enabling a mutual solute–solvent polarization treatment [40,42]. Both frameworks are particularly suitable for systems in polar protic media, where specific interactions, e.g., hydrogen bonding, are prominent [43,44,45,46,47,48]. Moreover, these fully atomistic models can be combined with a dynamical sampling of the solute–solvent phase space [40], typically through classical MD (with more sophisticated sampling options also possible [49]), thus explicitly accounting for the dynamical aspects of solvation.

We here apply our protocol to two organic dyes: 7-methoxycoumarin and Nile red in aqueous environment. Both exhibit a pronounced sensitivity to the external environment [50,51,52,53,54,55,56,57,58,59,60], and their absorption features are strongly dependent on solvent polarity [61,62,63,64,65,66] and specific solute–solvent interactions, making them ideal benchmarks for testing explicit, polarizable QM/MM descriptions. 7-Methoxycoumarin provides a compact π-conjugated scaffold with a carbonyl group that can potentially engage in directional hydrogen bonding [67]. Similarly, Nile red is a rigid chromophore with strong visible-range absorption whose position and line shape depend markedly on solvent polarity and hydrogen-bonding patterns [62,63].

The paper is organized as follows. We first present numerical results, emphasizing conformational analysis and MD simulations to characterize conformations and hydrogen-bonding patterns. We then discuss the UV-Vis spectra. Next, we summarize the computational methodology for computing solution-phase absorption at the QM/FQ and QM/FQFμ levels and conclude with final remarks.

## 2. Results and Discussion

In this section, we discuss the optical response of 7-methoxycoumarin and Nile red in aqueous solution, as modeled at the QM/MM level. As outlined above, we employed two polarizable descriptions of water: (i) QM/FQ with three parameterizations (FQ^*a*^ from Ref. [68], FQ^*b*^ from Ref. [69], and FQ^*c*^ from Ref. [70]) and (ii) QM/FQFμ, in which we also enabled intermolecular charge transfer between solvent molecules (QM/FQF$\mu_{CT}$) [12,41].

### 2.1. 7-Methoxycoumarin in Aqueous Solution

**Figure 1:**
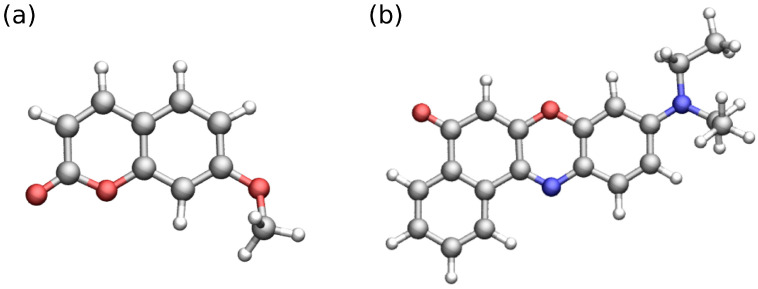
Molecular structures of (**a**) 7-methoxycoumarin and (**b**) Nile red.

7-methoxycoumarin is characterized by a main conformer in aqueous solution, as expected due to its relatively rigid molecular structure (see Figure 1) [67]. The molecule contains three oxygen atoms that could, in principle, participate in hydrogen bonding (HB) with water. Nonetheless, the analysis of the MD trajectories from Ref. [67] in terms of radial distribution function (RDF) indicates that only the carbonyl oxygen exhibits Oxygen-water distances characteristic of HB interactions.

**Figure 2:**
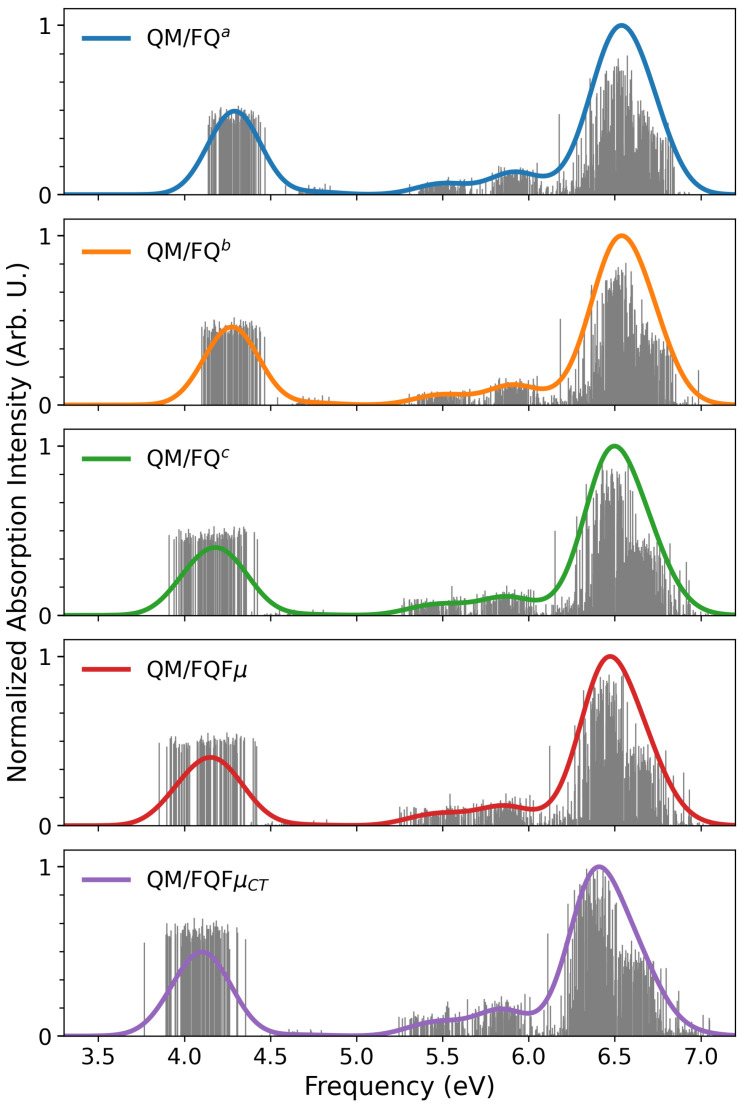
7-methoxycoumarin QM/FQ^*a*^, QM/FQ^*b*^, QM/FQ^*c*^, QM/FQFμ, and QM/FQF$\mu_{CT}$ UV-Vis raw data (sticks) together with their Gaussian convolution (FWHM = 0.3 eV).

In Figure 2, we report the computed stick spectra, i.e., the QM/MM raw transitions extracted from each snapshot for the five exploited methods (QM/FQ^*a*^, QM/FQ^*b*^, QM/FQ^*c*^, QM/FQFμ, and QM/FQF$\mu_{CT}$). The stick spectra clearly underscore the overall features of the final averaged spectrum which are obtained by a Gaussian convolution (full width at half maximum − FWHM = 0.3 eV). Such features arise from the snapshot-specific transitions, which reveal the dispersion of both wavelengths and intensities arising from the variation of the spatial arrangement of water molecules around the chromophore along the MD trajectory. It is worth remarking that the QM/MM inhomogeneous line shape emerges from our mixed dynamical-atomistic approach [40,45], which explicitly takes into consideration an ensemble of microscopically distinct configurations, each characterized by diverse spectroscopic responses. This stands in sharp contrast to implicit-solvent approaches such as QM/Polarizable Continuum Model (PCM) [16,71], which typically rely on one (or a few) minimum-energy structures and subsequently apply broad, empirical lineshape functions to mimic conformational and solvation disorder [17].

Overall, all methods deliver a qualitatively similar spectral pattern with two dominant bands: a high-energy band at ∼6.3–6.7 eV and a low-energy band at ∼4.0–4.5 eV, plus weaker shoulders in the 5.0–6.0 eV region. While the principal features remain consistent across the approaches, we note that QM/FQFμ, QM/FQF$\mu_{CT}$, and the QM/FQ^*c*^ parametrization yield a noticeably broader low-energy band. This behavior, also reported for other systems [43,72,73], is attributable to the enhanced electrostatic coupling between solute and solvent, which is obtained by exploiting the FQ^*c*^ parametrization and FQFμ force fields. Furthermore, we highlight that while the high-energy band shows only minor method-dependent shifts, the low-energy band undergoes a more appreciable displacement of ≈0.15 eV when moving from QM/FQ^*a*^ to QM/FQF$\mu_{CT}$, underscoring the different treatments of solute–solvent interactions offered by the two force fields and the various parameterizations [12].

**Figure 3:**
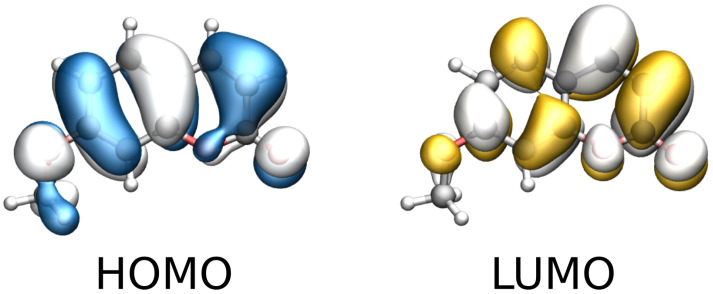
Graphical depiction of 7-methoxycoumarin HOMO and LUMO involved in the first bright transition (isovalue 0.02 a.u.).

To rationalize this finding, we examine the molecular orbitals (MOs) involved in the first transition, which are graphically depicted in Figure 3. The excitation has a clear $\pi \to \pi^{*}$ character and is dominated by a transition from an occupied MO to the lowest unoccupied MO (LUMO), consistent with a near-UV band at ∼290 nm. Remarkably, the carbonyl oxygen is directly involved in the excitation. Therefore, the marked sensitivity of the first band to the specific solvent model can be explained by considering that a strong, directional hydrogen bond is observed in the MD trajectory involving this atom [67]. Also, this explains that the large snapshot dispersion observed in the first band, which is accentuated when moving from QM/FQ^*a*^ to QM/FQFμ, naturally leads to the observed inhomogeneous broadening of the low-energy band.

**Figure 4:**
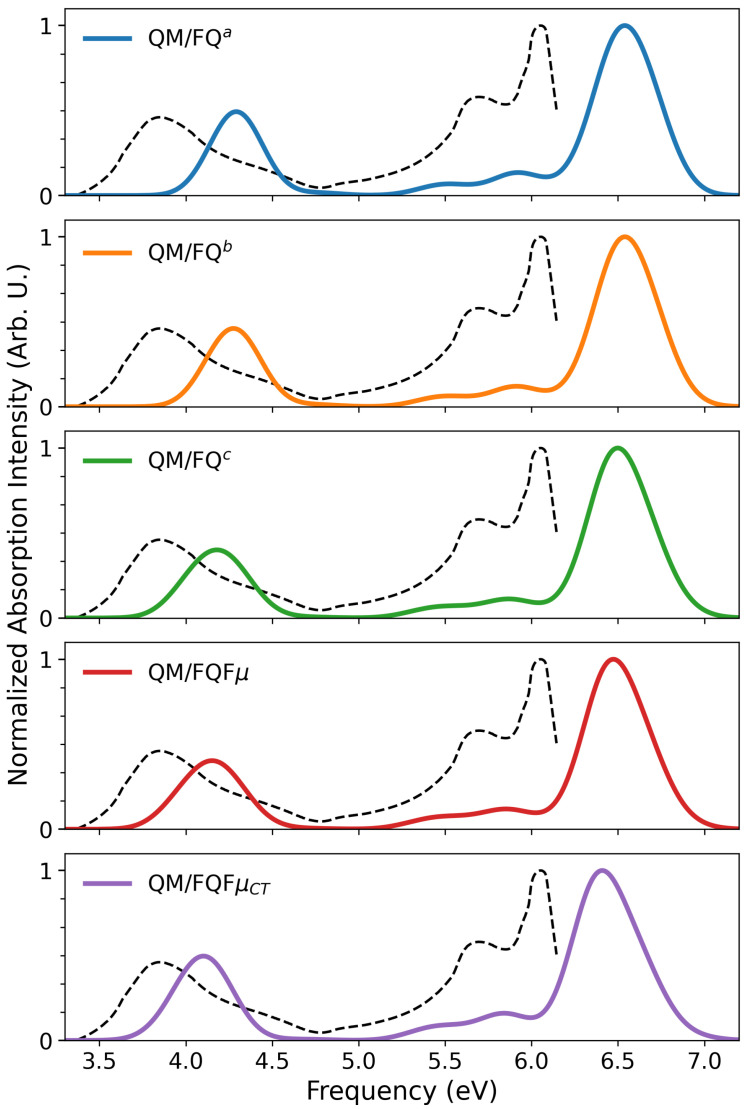
7-methoxycoumarin QM/FQ^*a*^, QM/FQ^*b*^, QM/FQ^*c*^, QM/FQFμ, and QM/FQF$\mu_{CT}$ computed UV-Vis spectra. The experimental spectrum from Ref. [74] is also depicted as a black dashed line.

We now compare with the experimental results reported in Ref. [74] (see Figure 4). The experimental spectrum presents a main peak at ∼6.0 eV, a shoulder at ∼5.7 eV, and a broad band near the visible region (around 3.8 eV). All the solvent models reproduce the overall spectral shape observed experimentally, although the shoulder near the main peak is merged into the principal band in every case. Notably, while a shift to higher energies is present due to the specific choice of DFT functional/basis set [75], the inclusion of the additional polarization sources in QM/FQFμ drives a progressive shift toward the experimental spectrum. The agreement is particularly good for QM/FQF$\mu_{CT}$. Moreover, all methods capture the relative intensity between the 6.0 eV main peak and the 3.8 eV band. A slight discrepancy is observed for QM/FQ^*b*^, QM/FQ^*c*^ and QM/FQFμ. However, an almost perfect agreement with the experimental relative intensities is reported for QM/FQ^*a*^ and QM/FQF$\mu_{CT}$.

### 2.2. Nile Red in Aqueous Solution

#### 2.2.1. The MD Analysis and Hydrogen Bonding Patterns

**Figure 5:**
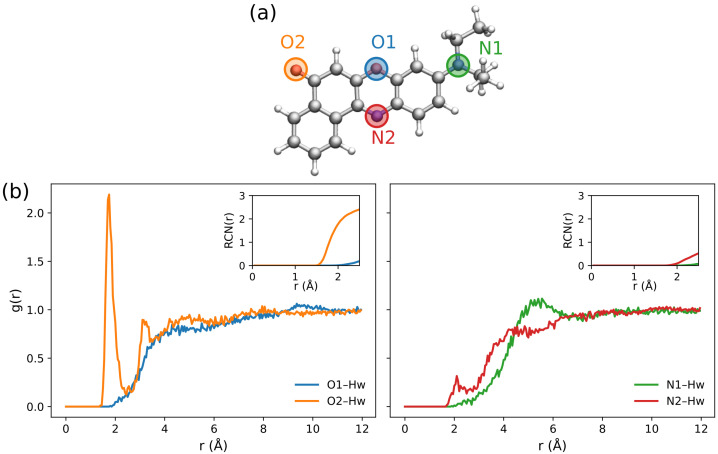
(**a**) Molecular structure and atom labeling of Nile red and (**b**) Radial distribution functions of O1, O2, N1, and N2 (see panel (**a**)) interacting with the hydrogen atoms of the water solvent (Hw).

Nile red is also characterized by a rigid structure with a dominant conformer in aqueous solution [63]. Nonetheless, it can form strong, directional hydrogen bonds with the water environment [63]. To analyze the hydrogen bonding patterns arising from the classical MD trajectory, analogously to the coumarin case, we compute the RDFs for the potential sites, namely the two oxygens (O1 and O2) and the two nitrogens (N1 and N2). The molecular structure and atom labels are shown in Figure 5a. All these sites can only act as potential hydrogen bonding acceptors; accordingly, in Figure 5b we report the RDFs between each site and the water hydrogens (Hw).

As seen in Figure 5b, the only dominant RDF peak corresponds to the O2–Hw interaction, involving, as expected, the carbonyl oxygen, whereas no significant peak is observed for the other atoms. For O2–Hw, the RDF maximum occurs at ∼1.8 Å, consistent with the hydrogen-bonding formation. To further characterize this interaction, we also computed the running coordination number (RCN) as a function of distance, which is the integral of the RDF up to *r*. This is shown in the insets of Figure 5b and indicates that, for the carbonyl–water interaction, two water molecules participate in the HB interaction, in line with the electronic structure of the oxygen atom in this specific functional group. Finally, we note that the N2–Hw RDF exhibits a small peak at ∼2.1 Å; however, the corresponding RCN confirms the absence of a specific hydrogen bond with the solvent for this site.

#### 2.2.2. Absorption Spectra

**Figure 6:**
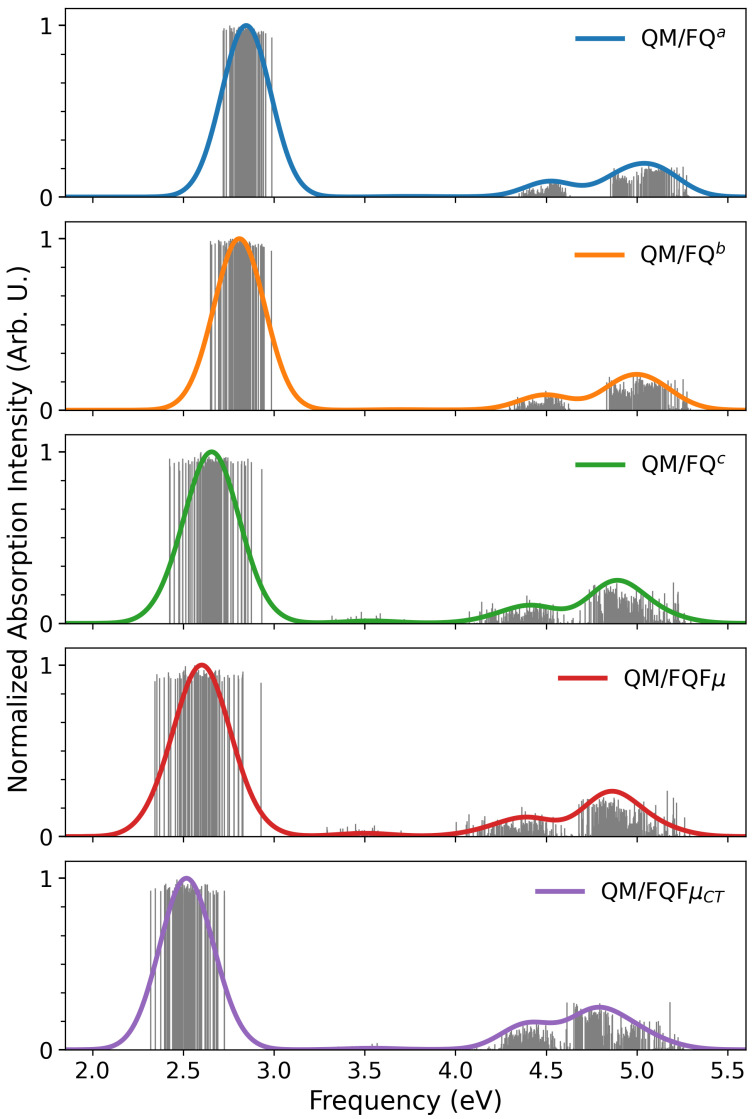
Nile red QM/FQ^*a*^, QM/FQ^*b*^, QM/FQ^*c*^, QM/FQFμ, and QM/FQF$\mu_{CT}$ UV-Vis raw data (sticks) together with their Gaussian convolution (FWHM = 0.3 eV).

In Figure 6 we report, the computed stick spectra for the five methods employed (QM/FQ^*a*^, QM/FQ^*b*^, QM/FQ^*c*^, QM/FQFμ, and QM/FQF$\mu_{CT}$), the computed stick spectra. As for coumarin, the stick spectra clearly encode the main features of the final averaged spectrum, obtained by Gaussian convolution (FWHM = 0.3 eV). These features again arise from snapshot-specific transitions, which capture the dispersion of both wavelengths and intensities caused by variations in the spatial arrangement of water molecules around the chromophore along the MD trajectory. All methods yield a qualitatively similar pattern, with a characteristic low-energy band at ∼2.5–2.9 eV and two broader high-energy bands at ∼4.0–5.0 eV. Across the exploited methods, the main spectral features are consistent. However, QM/FQFμ and QM/FQ^*c*^ exhibit a noticeably broader low-energy band. This behavior, also reported for coumarin, is attributable to the enhanced electrostatic coupling between solute and solvent afforded by the FQ^*c*^ parametrization and the FQFμ force field. In contrast to the previous case, we note that QM/FQF$\mu_{CT}$ predicts a significantly less broad low-energy band than QM/FQFμ, underscoring a different depiction of solute–solvent interactions in the two approaches, which also results in a redshift of the band for QM/FQF$\mu_{CT}$. This is also reported for the other bands: the entire spectrum, in fact, shifts toward lower energies when moving from QM/FQ^*a*^ to QM/FQF$\mu_{CT}$. However, as for coumarin, the most pronounced shift (≈0.25 eV) occurs for the low-energy UV–Vis band.

**Figure 7:**
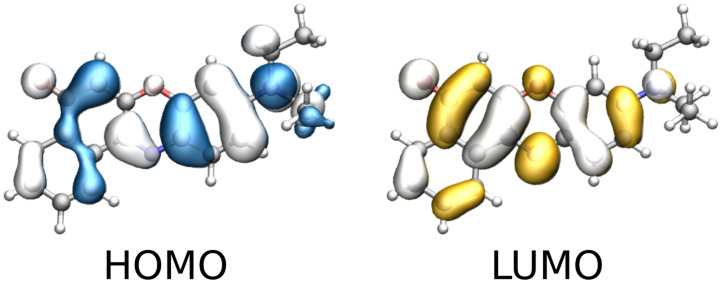
Graphical depiction of Nile red HOMO and LUMO involved in the first bright transition (isovalue 0.02 a.u.).

To rationalize this finding, we inspect the MOs involved in the first transition, which are depicted in Figure 7. The excitation has a clear $\pi \to \pi^{*}$ character and is dominated by a delocalized HOMO-LUMO transition, consistent with a visible band which falls at about 500 nm. Again, the carbonyl group is involved in the transition, and is directly involved in a strong, directional hydrogen bond as sampled in the MD trajectory (see Figure 5b). Therefore, the first transition band reports the most pronounced sensitivity to the solvent model.

**Figure 8:**
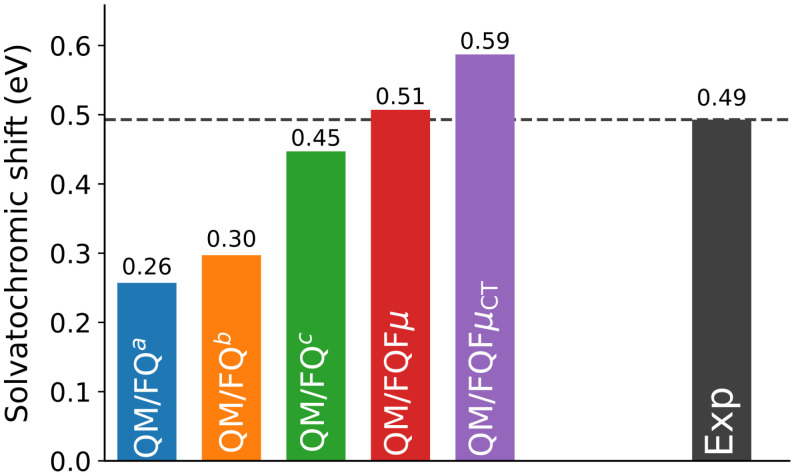
Nile red QM/FQ^*a*^, QM/FQ^*b*^, QM/FQ^*c*^, QM/FQFμ, and QM/FQF$\mu_{CT}$ computed vacuo-to-water solvatochromic shifts. The experimental value from Ref. [62] is also reported.

We compare our results with the experiment (see also Figure S3 in the Supplementary Material). As outlined in the Introduction, Nile red is among the most studied dyes for its pronounced solvent sensitivity, which leads to marked solvatochromism, i.e., a change in color upon varying the solvent [2,3]. Accordingly, we evaluate solvatochromic shifts (ΔE) as [76]:

(1) $$\Delta E=E_{\mathrm{vac}}-E_{\mathrm{solv}}$$

where $E_{\mathrm{vac}}$ and $E_{\mathrm{solv}}$ are the absorption energies of the first bright excitation of Nile red in the gas phase and in solution, respectively. In this work, we consider vacuo-to-water solvatochromic shifts. Since Nile red undergoes a red shift when moving from vacuum to solvent, the corresponding ΔE values computed using Equation (1) are positive. The experimental reference, extracted from Ref. [62], is ΔE=0.49 eV (gas-phase absorption at 480 nm; aqueous solution at 593.2 nm). The computed values are referenced to the gas-phase absorption maximum predicted at 3.10 eV at the same level of theory (see Section 3). The computed and experimental results are shown as a bar plot in Figure 8 (see also Figure S3 in the Supplementary Material).

We clearly observe that the two FQ parameterizations designed for reproducing water bulk properties [68,69], QM/FQ^*a*^ and QM/FQ^*b*^, do not reproduce the experimental reference, exhibiting substantial errors of about 0.24 and 0.19 eV, respectively. The agreement improves markedly for the parametrizations and methods tailored to capture solute-solvent electrostatic and polarization interactions, namely QM/FQ^*c*^, QM/FQFμ, and QM/FQF$\mu_{CT}$. In particular, both QM/FQ^*c*^ and QM/FQFμ predict the solvatochromic shift with errors within chemical accuracy (1 kcal/mol; 0.04 eV), with QM/FQFμ almost perfectly matching the experiment with an overestimation of only 0.02 eV. Among the selected methods, only QM/FQF$\mu_{CT}$ predicts a noticeably larger shift than experiment by 0.10 eV. It is worth noting that incorporating a quantum treatment of Pauli repulsion at the QM/MM level is expected to improve the agreement for QM/FQF$\mu_{CT}$ because such interaction acts in an opposite direction as electrostatics and polarization [12,76]. A slight deterioration could instead occur for all other models. Overall, these results highlight the high quality that can be achieved by using the polarizable QM/FQ and QM/FQFμ, provided a reliable parametrization of solute–solvent interactions is employed.

## 3. Materials and Methods

To compute the UV-Vis absorption spectra of solvated systems within QM/MM, we follow the workflow outlined in Refs. [40,45]. This protocol is defined by a sequence of steps designed to consistently capture the relevant physico-chemical interactions between the solute and its environment and to propagate them into the calculated spectra.

**Figure 9:**
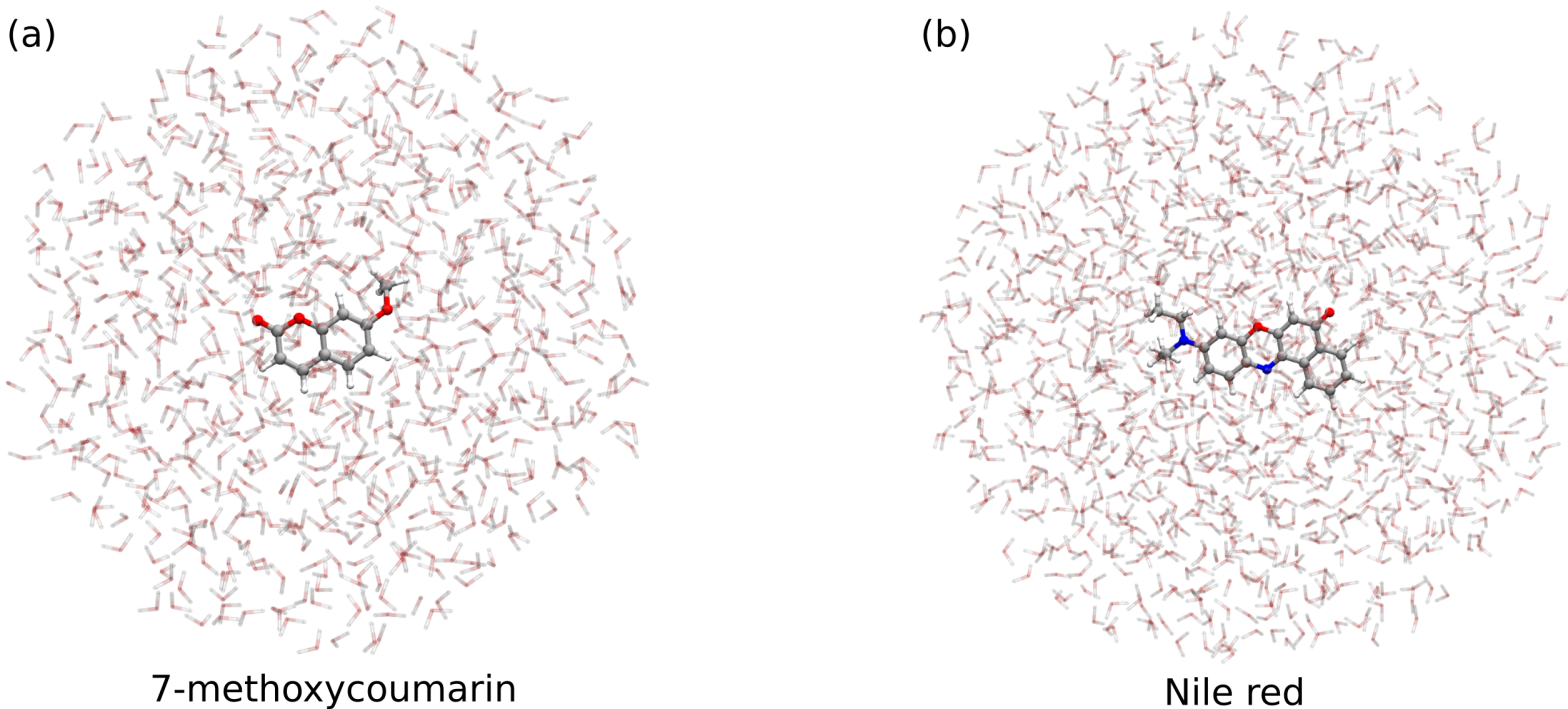
Graphical representation of (**a**) 7-methoxycoumarin and (**b**) Nile red snapshots.

1. *Definition of QM/MM partitioning:* The system must be partitioned into “solute” and “solvent” regions. In this work, the solute is described at the QM level, whereas the solvent is modeled at the MM level.
2. *Classical MD simulation:* To capture the dynamical aspects of solvation, the solute–solvent phase space must be sampled. In this work, we exploit classical, non-polarizable MD simulations, which are particularly reliable when combined with QM/MM for computational spectroscopy [40]. For 7-methoxycoumarin in water, we exploit the trajectories of Ref. [67], where a 100 ns classical MD simulation was carried out.
  For Nile red in water, intramolecular and intermolecular interactions are modeled with the OPLS-AA force field [77]. Solute and solvent bonded/non-bonded parameters are generated with antechamber [78,79], except for water, which is modeled by TIP3P [80]. Atomic charges for both solute and solvent are obtained *via* the RESP procedure [81] at the CAM-B3LYP/6-311+G*/PCM level. During MD, the solute is constrained at its minimum-energy geometry optimized at CAM-B3LYP/6-311+G*/PCM. The solute is then fully solvated in TIP3P water in a cubic box of edge ∼93.7 Å (26,853 water molecules) under periodic boundary conditions. The temperature is maintained constant at 300 K using the velocity rescaling method (coupling constant 0.1 ps) [82]. Electrostatics is treated with particle-mesh Ewald [83] with a 1.0 nm real-space cutoff; the same cutoff is also used for van der Waals interactions. Each system is equilibrated by a 1 ns NPT run (Berendsen barostat, 2.0 ps coupling) [84], followed by a 10.5 ns NVT production run to sample the configuration space. All simulations were performed with GROMACS [85].
3. *Extraction of structures:* A total of 100 uncorrelated snapshots are extracted from each MD trajectory for the subsequent QM/MM calculations. Each snapshot is cut in a spherical shape with a radius of 17 Å for 7-methoxycoumarin and 20 Å for Nile red (see Figure 9). The number of snapshots is selected to ensure convergence of the final average spectrum (see Figures S1 and S2 in the Supplementary Materials).
4. *Polarizable QM/MM calculations*: We compute the absorption spectrum of each extracted snapshot at the fully polarizable QM/FQ and QM/FQFμ levels. Within QM/MM, the total energy can be written as the following [28]:
  (2) $$E=E_{\mathrm{QM}}+E_{\mathrm{MM}}+E_{\mathrm{QM}/\mathrm{MM}}^{\mathrm{int}}$$
  where $E_{\mathrm{QM}}$ and $E_{\mathrm{MM}}$ are the energies of the QM and MM regions, and $E_{\mathrm{QM}/\mathrm{MM}}^{\mathrm{int}}$ is their interaction energy. In QM/FQ and QM/FQFμ, the MM sites are endowed with fluctuating charges (FQ) and additional fluctuating dipoles (FQFμ) that adapt to the QM density $\rho_{\mathrm{QM}}$. The QM/MM interaction can then be expressed as [40,41,45]:
  (3) $$\begin{array}{cc} E_{QM/FQ} & =\sum_{i}^{N_{q}}q_{i}\left(\rho_{\mathrm{QM}}\right)V_{i}\left(\rho_{\mathrm{QM}}\right) \end{array}$$
  (4) $$\begin{array}{cc} E_{QM/FQF\mu } & =\sum_{i}^{N_{q}}q_{i}\left(\rho_{\mathrm{QM}}\right)V_{i}\left(\rho_{\mathrm{QM}}\right)-\sum_{j}^{N_{\mu }}\mu_{j}\left(\rho_{\mathrm{QM}}\right)E_{j}\left(\rho_{\mathrm{QM}}\right) \end{array}$$
  where $q_{i}$ and $\mu_{j}$ are the charge on site *i* and dipole on site *j*, while $V_{i}\left(\rho_{\mathrm{QM}}\right)$ and $E_{j}\left(\rho_{\mathrm{QM}}\right)$ are the QM electrostatic potential and field evaluated at the corresponding MM sites. Charges and dipoles, polarized by $\rho_{\mathrm{QM}}$, are obtained by solving the linear system [45]
  (5) $$\begin{array}{cc} \left(\begin{array}{ccc} T^{qq} & 1_{\lambda } & T^{q\mu }\\ 1_{\lambda }^{†} & 0 & 0\\ T^{q\mu^{†}} & 0 & T^{\mu \mu } \end{array}\right)\left(\begin{array}{c} q\\ \lambda \\ \mu \end{array}\right)\; & =\left(\begin{array}{c} -\chi \\ Q_{\mathrm{tot}}\\ 0 \end{array}\right)+\left(\begin{array}{c} -V(D)\\ 0\\ E(D) \end{array}\right) \end{array}$$
  where $1_{\lambda }$ collects the Lagrange-multiplier blocks enforcing that the total charge on each MM molecule is fixed (or the total system charge in FQF$\mu_{CT}$), and $T^{qq}$, $T^{q\mu }$, and $T^{\mu \mu }$ are the charge–charge, charge–dipole, and dipole–dipole interaction kernels. These kernels depend on atomic chemical hardnesses η and polarizabilities α, while electronegativities χ enter on the right-hand side. Together with η and α, these parameters define the FQ (χ,η) and FQFμ (χ,η,α) force fields. Note that the FQ linear system is recovered from Equation (5) by removing the dipole-related rows and columns.
  Absorption spectra are obtained by using linear-response time-dependent DFT (TDDFT) [86] coupled to QM/FQ and QM/FQFμ (see Refs. [43,45]). In this framework, MM polarization sources respond self-consistently to the QM transition density, providing a consistent treatment of polarization in the linear-response regime. All polarizable QM/MM calculations were performed with a locally modified version of Gaussian 16 [87]. The QM region is treated with CAM-B3LYP [88] combined with the 6-311+G* basis set for 7-methoxycoumarin (following Ref. [67]) and 6-31+G* for Nile red. We request ten excited states for each TDDFT calculation. Solvent molecules in the MM region are described with three FQ parameter sets, taken from Refs. [68,69,70] and with FQFμ parameters extracted from Ref. [41].
5. *Extraction of spectra, and comparison with experiments*: The spectrum of each snapshot is extracted and ensemble-averaged to obtain the final profiles. Each spectrum is convolved with a Gaussian function (FWHM = 0.3 eV). The resulting computed spectra are then compared against the available experimental data.

## 4. Conclusions

In this work, we presented a computational study on the UV-Vis absorption spectra of two organic dyes, 7-methoxycoumarin and Nile red, dissolved in aqueous solution. Our protocol combined classical MD simulations to sample the solute–solvent phase space, with polarizable QM/MM embedding (QM/FQ using three parameterizations and QM/FQFμ, including the CT-enabled variant QM/FQF$\mu_{CT}$) to model mutual solute–solvent interactions. In this way, inhomogeneous broadening and the distribution of local environments emerged naturally from the ensemble. For 7-methoxycoumarin, the spectral features obtained at the various levels reflect the underlying solute–solvent interactions and dynamics. In particular, the broader low-energy band predicted by QM/FQ^*c*^, QM/FQFμ, and QM/FQF$\mu_{CT}$ is consistent with the stronger electrostatic coupling predicted by such methods, given that the frontier MOs involved in the transition include the carbonyl oxygen, which is involved in a strong, directional HB. Overall, all models reproduced the experimental profile. For Nile red, the MD analysis indicated that hydrogen bonding patterns were dominated by the carbonyl–water interaction. All approaches yielded a common qualitative spectral profile characterized by a low-energy band at ∼2.5–2.9 eV and broader bands at 4.0–5.0 eV. The computed vacuo-to-water solvatochromic shifts showed that bulk-oriented parameterizations (QM/FQ^*a*^, QM/FQ^*b*^) underestimated the experimental reference, whereas QM/FQ^*c*^ and QM/FQFμ achieved errors within chemical accuracy, with QM/FQFμ almost perfectly reproducing the experiment. QM/FQF$\mu_{CT}$ overestimated the shift by ∼0.10 eV. However, given the opposing role of short-range repulsion with respect to polarization, introducing an explicit QM/MM Pauli term is expected to improve this specific case.

Overall, our results demonstrate that faithful sampling of configuration space and a physically grounded, polarizable embedding are jointly essential to achieve good agreement with experiment for diverse chromophores. Our analysis also connected specific structural motifs (carbonyl centered HBs) to spectral trends (band shifts and broadening). In future work, we will pursue a more complete quantum treatment of short-range interaction (Pauli/dispersion at the QM/MM boundary) [12,89,90,91,92]. Extending the study to additional solvents and to emission processes [93] will further test the robustness and predictive power of the QM/FQ and QM/FQFμ frameworks also by exploiting highly correlated [92] and multi-reference wavefunctions [72] for describing the QM portion, provided that reliable parameterization and accurate dynamical sampling are employed [76].

## Data Availability

All the data are provided within the paper.

## Supplementary Materials

The following supporting information can be downloaded at the website of this paper posted on https://www.mdpi.com/article/10.3390/molecules30244675/s1; Figures S1 and S2. convergence of the QM/MM spectra as a function of the number of snapshots. Figure S3. Comparison of Nile red computed and experimental spectra [94].
