# Supplementary material for: The UV-Visible Absorption Spectra of Coumarin and Nile Red in Aqueous Solution: A Polarizable QM/MM Study
Source: Molecules. 2025 Dec 5;30(24):4675. doi: 10.3390/molecules30244675 (PMC12736338; doi:10.3390/molecules30244675)
Supplement: Supplementary file 1 [file molecules-30-04675-s001.zip › molecules-3990962-supplementary.pdf]

Supplementary Materials: The UV-Visible Absorption Spectra of Coumarin and Nile Red in Aqueous Solution: A polarizable QM/MM study

Tommaso Giovannini,*,† Matteo Ambrosetti,‡ and Chiara Cappelli‡

†*Department of Physics, University of Rome Tor Vergata, and INFN, Via della Ricerca Scientifica 1, 00133, Rome, Italy*

‡*Scuola Normale Superiore, Piazza dei Cavalieri 7, 56126 Pisa, Italy.*

E-mail: tommaso.giovannini@uniroma2.it

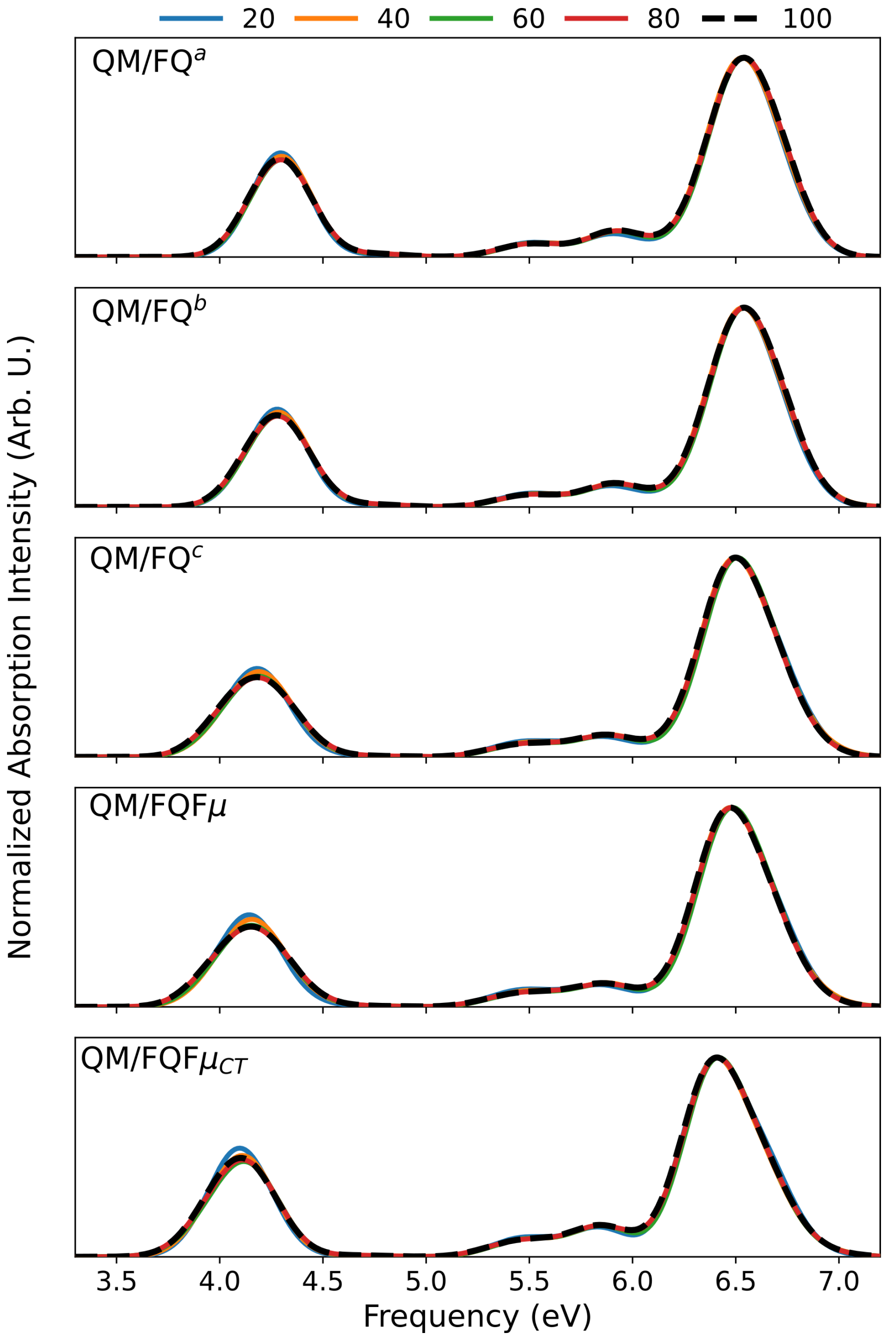

Figure S1: 7-methoxycoumarin QM/FQ^a, QM/FQ^b, QM/FQ^c, QM/FQFμ, and QM/FQFμ_{CT} UV-Vis spectra as a function of the number of snapshots, from 20 to 100.

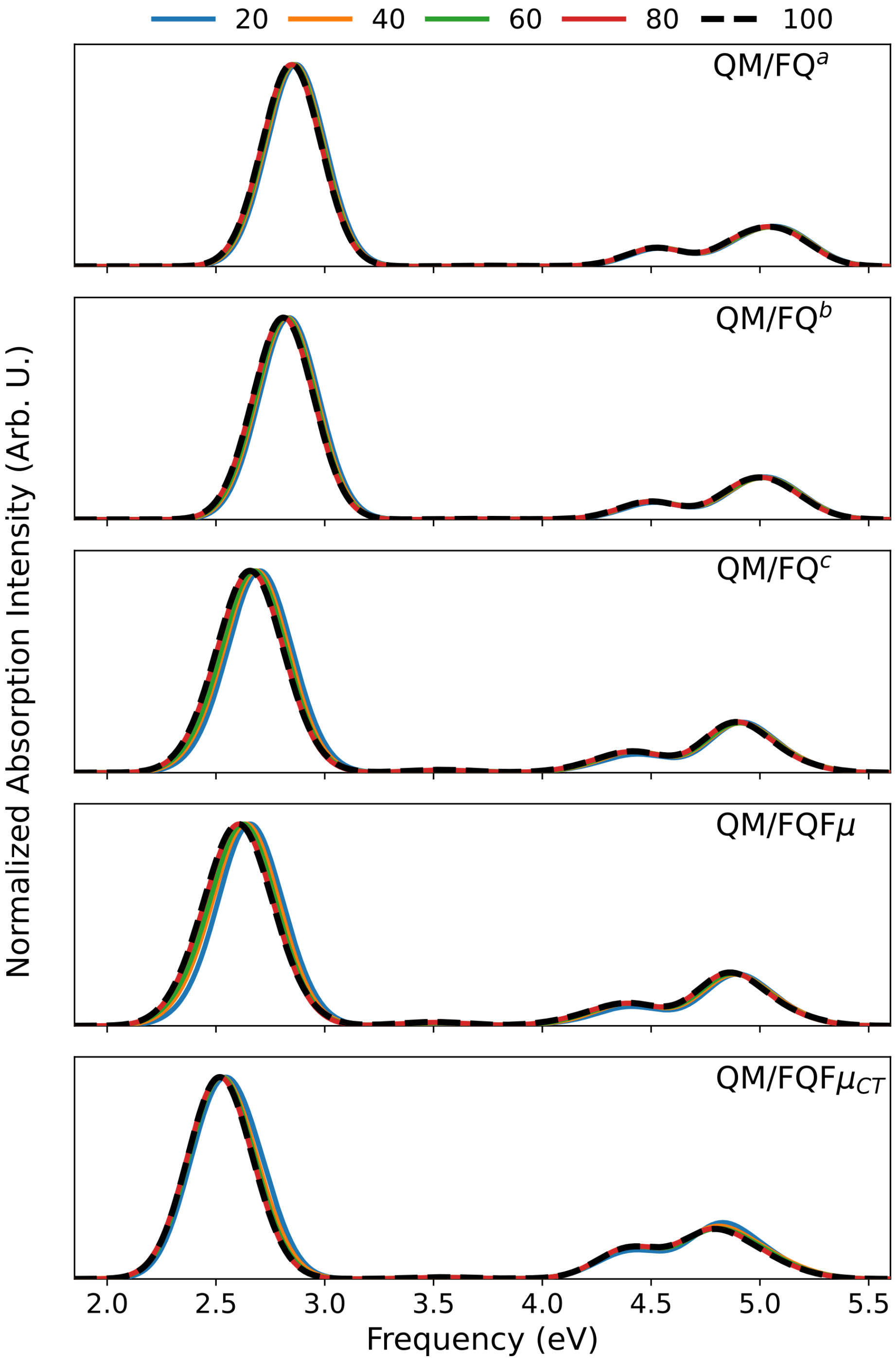

Figure S2: Nile red QM/FQ^a, QM/FQ^b, QM/FQ^c, QM/FQFμ, and QM/FQFμ_{CT} UV-Vis spectra as a function of the number of snapshots, from 20 to 100.

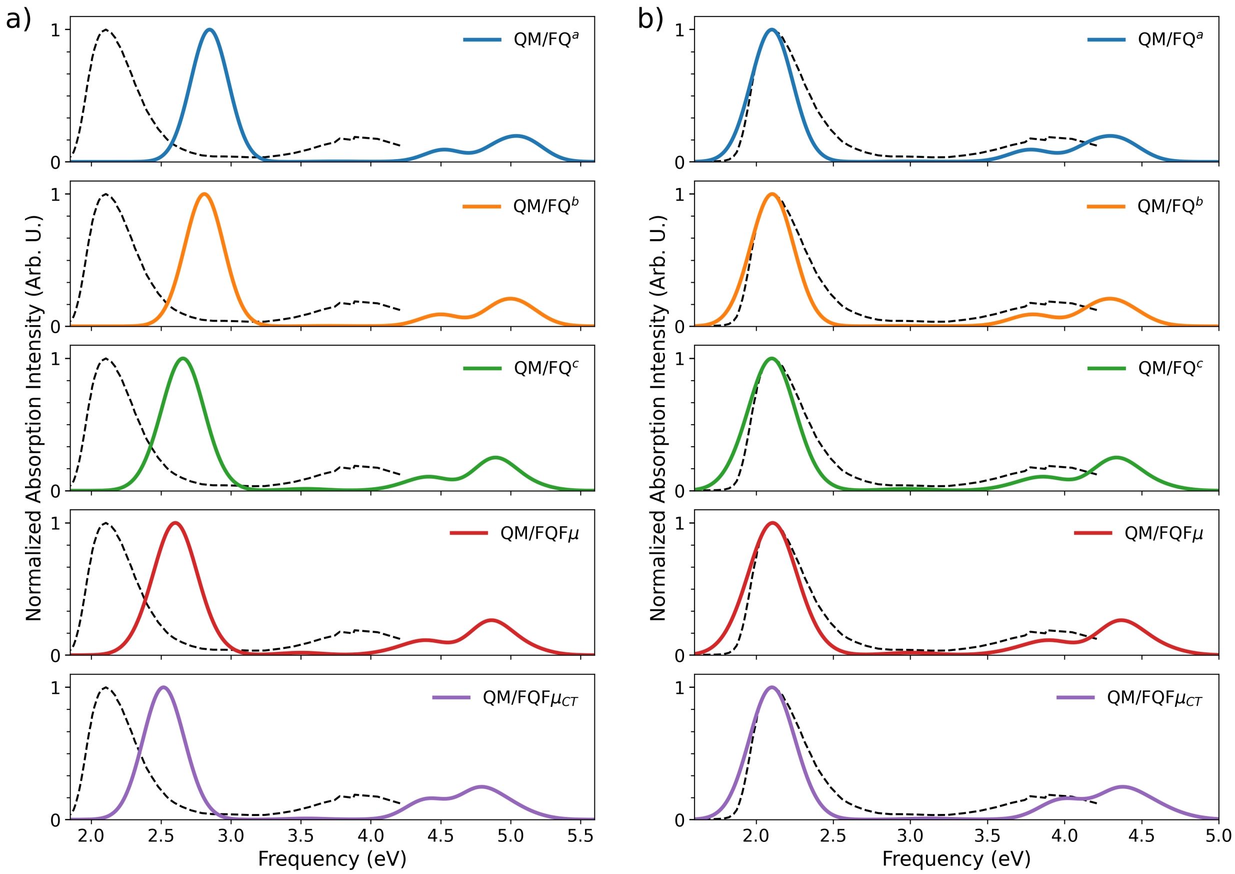

Figure S3: (a-b) Nile red QM/FQ^a, QM/FQ^b, QM/FQ^c, QM/FQFμ, and QM/FQFμ_{CT} computed UV-Vis spectra. The experimental spectrum from Ref. **?** is also depicted as a black dashed line. In (b) panel, the computed spectra are shifted to match the experimental main peak.
